# Laceration of the Flexor Metatarsi

**Published:** 1885-10

**Authors:** L. V. Plageman

**Affiliations:** Brooklyn, N. Y.


### II.—LACERATION OF THE FLEXOR METATARSI.
### (SUBCUTANEOUS.)

BY L. V. PLAGEMAN, M.R.C.V.S., BROOKLYN, N. Y.

---

On the 14th of March a bay gelding, about 9 years old, owned by an expressman, was brought to my infirmary, with symptoms resembling laminitis, as as far as action was concerned, but on closer examination and manipulation I diagnosed rheumatism, and treated accordingly (antiphlogistic). After a few days he became so lame in the off fore-leg that his owner thought he had fractured or dislocated it. Continued to treat him; gave salicylic acid and colchicum; in less than a week the lameness shifted to near fore-leg, and soon after to near hind one. After a few days more, probably a month, or less, from the first attack, the off hind-leg was the one where it rested. In about five weeks he was apparently sound, but I advised his owner not to use him; however, he did, and drove well for over a week; then, one day, he suddenly got lame in off hind-leg again, and on visiting him found a subcutaneous laceration of the flexor metatarsi—as I diagnosed it.

